# New Insights in RBM20 Cardiomyopathy

**DOI:** 10.1007/s11897-020-00475-x

**Published:** 2020-08-13

**Authors:** D. Lennermann, J. Backs, M. M. G. van den Hoogenhof

**Affiliations:** 1 Institute of Experimental Cardiology, Heidelberg University Hospital, Heidelberg, Germany; 2 DZHK (German Center for Cardiovascular Research), Partner Site Heidelberg/Mannheim, Heidelberg, Germany

**Keywords:** RBM20, Dilated cardiomyopathy, CaMKIIδ, Calcium handling, Gender differences, Titin

## Abstract

**Purpose of Review:**

This review aims to give an update on recent findings related to the cardiac splicing factor RNA-binding motif protein 20 (RBM20) and RBM20 cardiomyopathy, a form of dilated cardiomyopathy caused by mutations in *RBM20*.

**Recent Findings:**

While most research on RBM20 splicing targets has focused on titin (*TTN)*, multiple studies over the last years have shown that other splicing targets of RBM20 including Ca^2+^/calmodulin-dependent kinase IIδ (*CAMK2D*) might be critically involved in the development of RBM20 cardiomyopathy. In this regard, loss of RBM20 causes an abnormal intracellular calcium handling, which may relate to the arrhythmogenic presentation of RBM20 cardiomyopathy. In addition, RBM20 presents clinically in a highly gender-specific manner, with male patients suffering from an earlier disease onset and a more severe disease progression.

**Summary:**

Further research on RBM20, and treatment of RBM20 cardiomyopathy, will need to consider both the multitude and relative contribution of the different splicing targets and related pathways, as well as gender differences.

## Introduction

Dilated cardiomyopathy (DCM), as defined by left ventricular or biventricular systolic dysfunction and dilatation that are not explained by abnormal loading conditions or coronary artery disease, is a leading cause of death worldwide, and one of the main reasons for heart transplantation [1]. Its prevalence has long been thought to be around 1:2500 based on a study in Olmsted county (USA) published in 1989, but newer studies estimate it up to 1:250 [2, 3]. The European Society of Cardiology divides DCM into familial (genetic) and nonfamilial (non-genetic) forms, with 30–50% of cases being familial in nature [4, 5]. In contrast to hypertrophic cardiomyopathy (HCM) and arrhythmogenic right ventricular cardiomyopathy (ARVC), where a small number of genes account for most of the genetic causes, DCM-causing mutations have been observed in a variety of genes of diverse ontology [2]. One of these genes is *RBM20*, of which mutations have been observed in 2–3% of familial DCM cases, and which interestingly has been shown to account for a number of DCM cases for which previously no genetic cause could be established [5–8]. The *RBM20* gene encodes RNA binding motif protein 20, a *trans*-acting splicing factor [7]. RBM20 is highly expressed in striated muscle, especially the heart, and has been shown to affect splicing of a variety of genes important for cardiac function [7, 9, 10]. RBM20 cardiomyopathy manifests as a highly penetrant and aggressive disease which is correlated with high rates of heart failure, arrhythmias, and sudden cardiac death [7, 11•, 12, 13]. The most frequently investigated splicing target of RBM20 is *TTN*, which encodes the giant sarcomeric protein titin. It is important to note that mutations in the *TTN* gene itself are also frequent causes of DCM, accounting for ~ 20% of familial and ~ 15% of sporadic DCM cases [5, 14]. In 2012, Guo and colleagues described the first Rbm20-deficient animal model, namely a rat model with an unusually large titin protein, which they found to be due to a naturally occurring deletion in the *Rbm20* gene [9]. Therefore, because *TTN* was among the first genes shown to be regulated by RBM20, its connection to DCM, and its importance in cardiac biology, the research focus on RBM20 cardiomyopathy mostly lay on *TTN*. However, recent findings suggest that RBM20 cardiomyopathy cannot solely be explained by changes in *TTN* splicing, and that missplicing of additional RBM20 target genes is likely to contribute to the phenotype [11•]. Currently, over 30 genes have been shown to be a target of RBM20 (Table 1). Among them are further genes important for sarcomeric function like *LDB3* and *TNNT2*, as well as many genes crucial for calcium handling in cardiomyocytes. One example is the calcium/calmodulin-dependent protein kinase II (CaMKII), which modulates excitation-contraction coupling by phosphorylating targets like the ryanodine receptor (RyR), phospholamban (PLN), and the L-type Ca^2+^ channel, some of which are also targets of RBM20 [15]. Rbm20 deficiency has been shown to lead to altered cellular calcium handling, signified by a cellular calcium overload [11•]. This may be caused by the altered splicing of *Camk2d* towards the CaMKIIδA and CaMKIIδ9 isoforms, as other studies showed that an increase in CaMKIIδA is sufficient to cause similar calcium handling defects in mice [16]. Remarkably, in these models, gender differences were observed, with male mice being more affected [16]. Likewise, male patients with RBM20 cardiomyopathy also show a more severe disease phenotype [17•]. Taken together, these findings imply that misspliced CaMKIIδ might critically contribute to the RBM20 cardiomyopathy phenotype, which would explain pathological calcium handling, arrhythmias, and sudden cardiac death, and might also be responsible for the observed gender differences. In this review, we aim to summarize these recent insights in RBM20 cardiomyopathy, and provide possible new translational approaches based on these new insights.

**Table 1:** Known targets of RBM20

Gene name	Protein	Function	References
APTX	Aprataxin	DNA repair	[9]
CACNA1C	Calcium channel, voltage-dependent, L-type, alpha 1C sub-unit	Sub-unit of the L-type calcium channel	[9]
CAMK2D	Calcium/calmodulin-dependent protein kinase II delta	Serine/threonine kinase; regulates many cardiac proteins through phosphorylation	[9, 11•]
CAMK2G	Calcium/calmodulin-dependent protein kinase II Gamma	Serine/threonine kinase; regulates many cardiac proteins through phosphorylation	[9]
DAB1	Disabled-1	Neuronal development	[9]
DNM3	Dynamin-3	Actin-membrane budding	[9]
DST	Dystonin	Adhesion junction plaque protein	[21]
DTNA	Dystrobrevin alpha	Part of the dystrophin-assoicated complex linking ECM and cytoskeleton	[9]
ENAH	Protein-enabled homolog	Actin-associated	[21]
FHOD3	Formin homology 2 domain-containing 3	Sarcomeric assembly	[9]
FNBP1	Formin-binding protein 1	Actin cytoskeleton regulation	[9]
GIT2	G protein-coupled receptor kinase interactor 2	Cytoskeletal dynamics	[9]
IMMT	Inner membrane mitochondrial protein	Part of the mitochondrial inner membrane complex	[21]
KALRN	Kalirin	Serine/threonine protein kinase	[9]
KCNIP2	*K*_*V*_ channel-interacting protein 2	Sub-unit of voltage-gated potassium channel complex	[9]
LDB3	LIM domain binding 3	Sarcomeric stabilization	[9, 21]
LMO7	LIM domain only protein 7	-	[21]
LRRFIP1	Leucine-rich repeat flightless-interacting protein 1	Transcriptional repressor	[21]
MECP2	Methyl CpG–binding protein 2	Transcriptional regulator; highly expressed in neuronal cells	[9]
MLIP	Muscular-enriched A-type laminin-interacting protein	Interacts with lamin A/C; potentially involved in cardiac homeostasis	[21]
MTMR1	Myotubularin-related protein 1	-	[9]
MYH7	Myosin heavy chain 7	Cardiac slow twitch myosin heavy chain beta isoform; muscle contraction	[21]
MYOM1	Myomesin-1	Sarcomeric; links titin and thick filament	[21]
NEXN	Nexilin	Actin-associated; DCM-associated	[21]
NFIA	Nuclear factor I A	Transcription factor	[9]
NPRL3	Nitrogen permease regulator-like 3	Inhibits mTORC1; necessary for cardiovascular development	[9, 100]
NTRK3	Tropomyosin receptor kinase C	Neutrophin-3-receptor	[9]
OBSCN	Obscurin	Sarcomeric signaling	[21]
PDLIM3	PDZ and LIM domain protein 3	Binds alpha actinin-2; relevant for right ventricular function	[21]
PDLIM5	PDZ and LIM domain protein 5	LIM domain protein; protein-protein interaction	[9, 101]
PLEKHA5	Pleckstrin homology domain-containing family A member 5	-	[9]
RALGPS1	Ral GEF with PH domain- and SH3-binding motif 1	-	[9]
RTN4	Reticulon 4	Neurite outgrowth inhibitor in the central nervous system	[21]
RYR2	Ryanodine receptor 2	Calcium receptor in the SR; allows release of Ca^2+^ into the cytosol	[21]
SEMA6D	Semaphorin 6D	Neuronal regulation	[9]
SH3KBP1	SH3 domain-containing kinase-binding protein 1	-	[9]
SLC38A10	Putative sodium-coupled neutral amino acid transporter 10	Sodium-dependent amino acid/proton antiporter	[9]
SORBS1	Sorbin and SH3 domain-containing 1	Cytoskeletal formation	[9]
SPEN	Msx2-interacting protein	Hormone inducible transcriptional repressor	[9]
TNNT2	Cardiac troponin T	Part of the cardiac troponin complex regulating muscle contraction dependent on calcium	[21]
TPM1	Tropomyosin alpha-1 chain	Cytoskeletal; contraction	[9]
TRDN	Triadin	Forms a complex with RyR and CASQ2; calcium release from the SR	[9]
TTN	Titin	Sarcomeric spring; compliance of the heart	[9, 21]
UBE2F	Ubiquitin-conjugating enzyme E2 F (putative)	-	[9]
ZNF451	E3 SUMO-protein ligase ZNF451	Protein sumoylation	[9]

Table showing gene and protein names of known targets of RBM20, as well as a short description of their main function. Genes with unknown or unclear function are marked with “-”

## Properties of *RBM20*

**Fig. 1:**
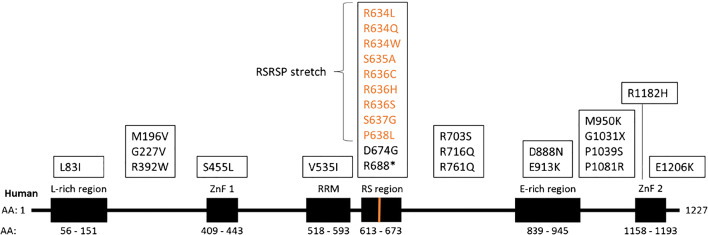
Schematic visualization of RBM20 protein structure, including functional domains. Known mutations shown above the corresponding domains. The RRM and RS region, especially the RSRSP stretch from amino acids 634–638 (marked in orange), are both important for RBM20’s nuclear localization. The RRM is required to bind to the “UCUU” RNA sequence found on targets of RBM20. RBM20’s regulatory function is likely achieved by the combined function of its functional domains. (ZnF, zinc finger domain; RRM, RNA recognition motif; RS region, serine-/arginine-rich region) (A single asterisk indicates a nonsense mutation) [6, 7, 12, 13, 17, 23, 68, 102–111]

The *RBM20* gene is located on chromosome 10, contains 14 exons, and encodes a protein of 1227 amino acids. The protein contains two zinc finger domains, an RNA recognition motif (RRM), a serine- and arginine-rich region (RS region), a leucine-rich region, and a glutamate-rich region (Fig. 1) [18]. These regions, especially the RS region, are highly conserved among species and mutations in these domains often cause a loss of RBM20 function [7, 12, 19]. RBM20 is predominantly located in the nucleus, and its nuclear localization is required for its function [10, 20]. The most frequent disease-causing RBM20 mutations occur in the RS region [19]. The serine and arginine residues in the RS region (RSRSP stretch) can be phosphorylated, which is necessary for the nuclear localization of Rbm20 [20]. The loss of phosphorylation in these mutated residues translocates RBM20 out of the nucleus, and this likely negates RBM20’s function [20]. The RS region also mediates protein-protein interactions with other splicing factors such as U2AF65 and U2AF35, potentially explaining the critical function of this domain for splicing activity [21]. The RS region, however, might not be the only region of RBM20 important for nuclear localization, as a truncated RBM20 peptide (including the RRM but not the RS region) is sufficient for nuclear localization [10]. This suggests that not the presence, but the phosphorylation status, of the RS domain determines nuclear localization. It would be interesting to see if a truncated peptide that includes the RRM, but also a mutated (and phospho-resistant) RS domain would localize to the cytoplasm.

Of a set of RBM20 mutants selectively lacking one of the zinc finger domains, the leucine-rich region, the RRM or the glutamate-rich region, only the loss of the glutamate-rich region led to a significant decrease in splicing activity [20]. Truncating RBM20 on the N-terminal part of the RS region, and thereby losing the glutamate-rich region, completely stopped splicing activity, although this cannot conclusively be explained by the loss of the glutamate-rich region [22]. Mutations of the glutamate-rich region, while not as frequent as mutations in the RS region, are also known to be associated with RBM20 cardiomyopathy [6, 23]. In one case, a mutation at amino acid 913 exchanging glutamate for lysine leads to a substantial reduction in RBM20 protein amount, which is hypothesized to be caused by misfolding of RBM20 due to the mutation [23]. It might be the case that the glutamate-rich region is more resistant to mutations affecting single amino acids compared with the RS region, as there is perhaps a greater degree of redundancy in the glutamate-rich region. The RRM also has functional relevance, as truncated RBM20 peptides without the RRM and the C-terminus of the protein lost all splicing function [22]. Selectively removing only the RRM however did not significantly reduce splicing function of RBM20, indicating that different regions of RBM20 affect its regulatory function [20]. Taken together, these data suggest that the different domains of Rbm20 have different functions, and that proper function of Rbm20 is an orchestrated process of most, if not all, of these domains. RBM20’s RRM binds to an RNA recognition element defined by a UCUU core, which most commonly is located up to 50 nucleotides upstream or 100 nucleotides downstream in the introns flanking an alternatively spliced exon [21, 24]. In the *TTN* pre-mRNA, RBM20 represses splicing of large stretches of exons, prohibiting the removal of flanking introns [25]. This allows alternative splice sites at the 3′ or 5′ end of RBM20-repressed regions to splice together, which can skip large numbers of exons and create different splice products depending on which alternate splice sites splice together [22, 25]. RBM20 also acts as a splicing repressor for many of its other splicing targets [21]. Interestingly, the interaction between RBM20 and *TTN* also affects the regulation of other RBM20 targets. In the nucleus, RBM20 is mainly localized in two speckles, which are the two transcriptional sites of the *TTN* gene [25]. During the transcriptional process, RBM20 colocalizes with the *TTN* pre-mRNA, which is simultaneously spliced [25, 26]. These clusters of *TTN* pre-mRNA and RBM20 are able to spatially attract the gene loci of other RBM20 targets like *CACNA1C* and *CAMK2D* [27•]. Remarkably, this colocalization leads to an increased splicing activity of RBM20 on these targets, and loss of *TTN* pre-mRNA leads to similar splicing changes in *CACNA1C* and *CAMK2D* as loss of RBM20 itself [27•]. These data suggest that the *TTN* pre-mRNA creates a RBM20 splicing factory, which is necessary for correct splicing of other RBM20 targets [27•]. RBM20 seems also able to generate circular RNA transcripts (circRNAs) from *TTN* and several of its other targets [28, 29]. In the case of *TTN*, RBM20-dependent exon skipping provides the substrate for back-splicing these skipped exons together to produce circRNAs. However, this accounts only for a small amount of cardiac circRNAs, while the vast majority is created in non-RBM20-dependent manner from constitutively spliced exons, competitively with the generation of the linear transcript [28]. Interestingly, RBM20 not only affects splicing but can also affect transcription of its targets, which might be related to its ability to generate circRNAs [30].

## Upstream Regulators of *RBM20*

In mice, *Rbm20* is induced during early cardiac development (Carnegie stage E8.5), and loss of Rbm20 leads to a dysregulation of cardiogenic genes [31]. The main pathway implicated in *Rbm20* regulation so far is the PI3K-Akt-mTOR axis, which was found to upregulate *Rbm20* expression [32, 33]. Activation of PI3K (phosphoinositide 3-kinase) causes Akt (also known as PKB; protein kinase B) to become phosphorylated, which in turn activates mTORC1. mTORC1 phosphorylates S6KI and 4E-BP1, which promote expression of *Rbm20* [32]. The PI3K-Akt-mTOR axis is activated by exercise and various growth factors, including insulin, insulin-like growth factor 1, and neuregulin, and its activation is cardioprotective in DCM [36, 37]. Akt especially has been shown to regulate other SR proteins in a growth factor–dependent manner [38]. From these growth factors, currently only insulin and the thyroid hormone T3 have been shown to have an effect on *Rbm20*, both leading to an upregulation of *Rbm20* expression [32, 33]. The connection to the thyroid hormones is interesting, since hypothyroidism is known to have adverse effects on heart function and correlates with disease severity in heart failure patients [39, 40]. In that regard, hypothyroidism could contribute to disease severity by regulating RBM20 expression and missplicing of downstream splicing targets, as it is known that RBM20 levels correlate with the amount of missplicing of its targets [21]. Another pathway connected to *Rbm20* is the mitogen-activated protein kinase (MAPK) pathway. Like the PI3K axis, it can be activated by growth factors like epidermal growth factor [41]. Angiotensin II has also been shown to activate this pathway, triggering its downstream target ETS transcription factor (ELK1), which binds to the *Rbm20* promotor and upregulates *Rbm20* expression [42]. The methylation status of *Rbm20* also affects its expression. In a study of rats treated with doxorubicin, which decreased the global amount of methylated DNA and specifically altered methylation in 7 different *Rbm20* nucleotide residues, *Rbm20* expression was increased almost twofold [43]. Interestingly, RBM20 methylation correlates with fasting insulin levels, although it is unknown if there is a functional relevance to this [44]. *RBM20* expression was also found to be positively correlated with BMI, and together these findings suggest an important influence of the body’s metabolic state on *RBM20* [45].

## Posttranslational Regulation of RBM20

Little is known about posttranslational regulation of RBM20, but it is likely that RBM20 is additionally regulated by posttranslational modifications (PTMs) such as phosphorylation, as other SR proteins have been shown to alter their localization, conformation, and splicing function if phosphorylated, and the phosphorylation of RBM20’s RS region has already been shown to be functionally relevant for splicing regulation [20, 34, 35]. Another PTM worth investigating may be ubiquitination of RBM20. In a study of a family with RBM20 cardiomyopathy caused by an E913K mutation in RBM20, protein levels of RBM20 were strongly reduced, while mRNA levels and stability were not, which may suggest that the new lysine functions as a substrate for ubiquitination, and leads to degradation by the proteasomal complex [23]. Further identification of PTMs of RBM20, and what enzymes/kinases are involved, will be valuable to unravel the mechanism(s) through which RBM20 functions, and could underlie new therapeutic approaches. One possible approach here would be the use of in silico predictions of the interactions of potential kinases with both wild-type and mutated forms of RBM20. Furthermore, RBM20-dependent splicing is regulated by polypyrimidine tract-binding protein 1 (PTBP1), also known as PTB4 or hnRNPI. PTBP1 belongs to a larger family of genes regulating mRNA and can also alter splicing [46–48]. The RRM of PTBP1 shares similarities in structure with RBM20’s RRM, and PTBP1 is able to bind simultaneously with RBM20 to the UCUU-binding motif shared by RBM20’s targets [22, 24]. Binding of PTBP1 counteracts RBM20’s effect on *TTN* splicing in a manner dependent on the ratio of PTBP1 to RBM20 [22]. Antagonistic mRNA regulation is also seen in other RNA-binding proteins; for example, the CELF and MNBL proteins regulate over 200 exons in an antagonistic manner [49]. However, PTBP1 and RBM20 do not only oppose each other, as splicing of formin homology 2 domain-containing 3 (FHOD3), a protein relevant for the assembly of the sarcomere, was changed towards an increase of FHOD3 isoform F both by addition of RBM20 and PTBP1 in a dose-dependent manner [50]. Examining the effect of PTBP1 on other known RBM20 targets and vice versa might lead to a better understanding of the interactions between these two factors.

## RBM20 Regulates Splicing of Sarcomeric Genes Including *TTN*

Many of RBM20’s known targets can be categorized as either sarcomeric, like *TTN*, *LDB3*, *TNNT2* (cardiac muscle troponin T), *MYH6*, and *MYH7* (myosin heavy chain alpha and beta isoform), or related to cellular ion handling, like *CAMK2D*, *RYR2*, *SLC8A1* (the sodium-calcium exchanger; also NCX), and *CACNA1C* (encoding the alpha 1C sub-unit of the L-type calcium channel; also Ca_V_1.2). The most frequently studied splicing target of RBM20 is *TTN*, but it should be noted that evidence that *TTN* is the only critical target of RBM20 is currently lacking. The *TTN* gene contains 363 exons, and its translational product (titin, also known as connectin) is the largest human protein, with a size of over 3 MDa. Titin connects the sarcomeric Z-disk and thick filaments in striated muscle, where it is among the most common proteins [51, 52]. It functions as a molecular spring, and together with collagen, titin is the main determinant of passive tension (as measured in demembranated cellular systems) in the sarcomere and thereby the muscle [51]. Cardiac titin mainly occurs in two isoforms which are co-expressed [53]. The smaller isoform is named N2B titin after the N2B element, an additional elastic segment found only in cardiac titin [54, 55]. The larger N2BA isoform additionally contains an elastic N2A element, as well as multiple elastic Ig-like and PEVK domains [54, 55]. This increase in size and the number of elastic elements in the N2BA isoform reduces the stiffness of this isoform compared to N2B titin [54, 55]. The ratio of these isoforms in a myocyte defines the cellular stiffness, with larger amounts of the larger N2BA isoform leading to myocytes with a reduced stiffness [55, 56]. Functionally, this results in an increased whole-heart compliance, as the cardiomyocytes react less rigidly to the force applied by the blood flow [55, 56]. In DCM and ischemic heart disease, the N2BA:N2B ratio is increased; however, it is unclear if this is a causative or compensatory effect [57–59]. A high percentage of DCM cases are caused by mutations leading to a truncated form of titin [5, 14]. However, in comparison to other forms of DCM, TTN cardiomyopathy represents a mild entity of DCM that becomes symptomatic at a higher age, has a better treatability, and relatively benign outcomes [60]. TTN cardiomyopathy is also diagnosed at a higher age when compared directly to RBM20 cardiomyopathy [61]. The loss of RBM20 function does not lead to truncation of the titin protein. Instead, the average titin protein becomes even larger—the amount of the smaller N2B isoform is reduced, while the larger N2BA isoform is upregulated and another, giant and even more compliant 3.9 MDa titin isoform called N2BA-G appears, which reduces the myocardial stiffness (as measured in intact cardiomyocytes/myocardial tissue) and increases resting sarcomere length [9, 23, 62–64]. Functionally, compliance of the heart increases, and passive tension of the sarcomere is reduced [23, 63]. Additionally, the Frank-Starling mechanism is impaired due to a diminished length-dependent activation (a reduction of the force generated by an increase in muscle length), which was reduced in both heterozygous and homozygous Rbm20-deficient mice [63, 65]. This results in a reduced fractional shortening (FS), and an even further reduced FS when the afterload is increased (for example through application of noradrenergic stress) [63]. Furthermore, the length-dependent Ca^2+^ sensitivity of the sarcomere is also affected by loss of RBM20 [23, 65]. A study in human RBM20-deficient cardiomyocytes found an increase in Ca^2+^ sensitivity, which could be counteracted by application of protein kinase A (PKA), while control cardiomyocytes showed no change after PKA application [23]. It is also known from other forms of heart failure that a reduction of PKA-mediated phosphorylation of sarcomeric proteins caused by a reduction of β-adrenergic signaling leads to an increase in Ca^2+^ sensitivity [112]. However, this is notably only found in human tissues, as in animal models the opposite (i.e., a reduction of Ca^2+^ sensitivity) is observed [65]. Some sarcomeric proteins are also phosphorylated by targets of RBM20; for example, CaMKII-dependent phosphorylation of titin is able to reduce the passive tension of the sarcomere [66]. The interplay of titin stiffness and calcium sensitivity also affects cross-bridge cycling, as loss of RBM20 causes a slowed cross-bridge detachment rate [65, 67]. Interestingly, a slowdown of cross-bridge detachment normally leads to an increased Ca^2+^ sensitivity, while the opposite is the case when RBM20 is lost [65, 67]. This may be explained by the altered titin, as titin plays a large part in modulation of the sarcomeric complex and interacts both with the thin and thick filament [65, 114]. Interestingly, heightened levels of collagen are observed in the RBM20-deficient heart, which may be a compensatory effect working against the reduced titin stiffness [63]. Together, these physiological changes likely contribute to the disease presentation of RBM20 cardiomyopathy. However, RBM20 cardiomyopathy shows a higher penetrance when compared with dilated cardiomyopathy in general, and with TTN cardiomyopathy specifically, with over 70% having a familial history of DCM and over 50% having a familial history of sudden cardiac death (SCD) [61, 11•]. Strikingly, RBM20 cardiomyopathy presents with a high prevalence of arrhythmias, especially sustained ventricular arrhythmia (VA) and tachycardia (VT), but also non-sustained VT and SCD, as well as high rates of atrial fibrillation [61, 11•]. This is in stark contrast to TTN cardiomyopathy [11•, 60]. Consequently, over 60% of RBM20 cardiomyopathy patients require implantation of an internal cardioverter defibrillator (ICD) compared with 27% of general DCM patients and < 10% of TTN cardiomyopathy patients [11•, 5]. RBM20 cardiomyopathy patients are also more likely to need a heart transplant compared to other DCM patients, and need it at a significantly younger age, with a mean of 28.5 years [8]. Together, these data suggest that the RBM20 cardiomyopathy phenotype is more severe and arrhythmogenic, and cannot be solely explained by changes in titin.

## RBM20 Regulates Splicing of Ion- and Calcium-handling Genes Including *CAMK2D*

Several studies have now investigated if and how loss of RBM20 can impact calcium handling. In a stem cell RBM20 knockdown model, the peak amplitude of calcium transients highly increased and the area under the curve (AUC) more than doubled, while the beating frequency went down significantly [31]. Two models of patient-derived pluripotent stem cell cardiomyocytes with mutations in the RS domain (S635A and R636S, respectively) showed similarly altered calcium handling [68–70]. Interestingly, application of norepinephrine stress increased these differences in peak amplitude and AUC, and additional application of either the calcium channel antagonist verapamil or the beta-blocker carvedilol could return these values to those of WT controls [70]. In a RBM20–KO mouse model, isolated cardiomyocytes showed an altered L-type calcium current (*I*_Ca,*L*_) density, which increased in amplitude almost twofold [11•]. In addition, these cardiomyocytes showed an intracellular calcium overload, signified by heightened diastolic Ca^2+^, heightened peak transient, and heightened SR calcium load, and they presented with an increased amount of pro-arrhythmic spontaneous calcium releases from the SR [11•]. The addition of norepinephrine increased the amount of spontaneous calcium releases, but they could be reduced to WT levels through treatment with verapamil [11•]. Verapamil is an inhibitor of the L-type calcium channel, so it seems likely that the increased L-type calcium current density is causative for the intracellular calcium overload and spontaneous calcium releases. The RBM20–KO mouse model also shows splicing differences in various ion handling genes, and it has been hypothesized that (some of) these underlie the calcium handling abnormalities [11•]. CaMKIIδ, for example, undergoes an almost complete switch from the normal δB and δC isoforms towards the δA and δ9 isoforms [11•]. CaMKIIδA was first described as a neurological isoform, but is also expressed during cardiac development up to 30 days after birth [16, 71]. Interestingly, its downregulation during cardiac development is dependent on RBM20 [31]. It is locally associated with the T-tubules and the intercalated discs, which are also the locations where calcium channels are highly concentrated [16]. In addition, a transgenic mouse model overexpressing specifically CaMKIIδA showed a similar phenotype to the one observed in RBM20 loss-of-function models, with increased SR calcium load and increased peak calcium transients [16]. Therefore, it has been hypothesized that CaMKIIδA could interact with the LTCC, and increase L-type calcium current [16, 11•]. CaMKIIδ9 is currently not well studied, but was recently found to be among the most abundant isoforms in the heart [72•]. Cardiac stress upregulates CaMKIIδ9 expression and hyper-activates it through phosphorylation and oxidation [72•]. CaMKIIδ9 promotes cardiomyopathy and cell death by phosphorylation of ubiquitin-conjugating enzyme E2T (UBE2T), an enzyme in the Fanconi anemia DNA repair pathway, which leads to degradation of UBE2T and thereby accumulation of DNA damage [72•]. Together, these results show that RBM20 cardiomyopathy is accompanied with an altered cellular calcium handling, which could be the reason for the heightened number of arrhythmias observed in patients. A possible mechanism for this could be increased activation of the LTCC through increased levels of CaMKIIδA, leading to a higher *I*_Ca,*L*_ and a cellular calcium overload; however, further research is required to validate this.

## Sex and Gender Differences in RBM20 Cardiomyopathy

Sex and gender differences in cardiac disease have gained attention over the last years. Different prevalence of risk factors, socioeconomic factors leading to differing utilization of health resources, and genetic differences between men and women lead to pronounced differences both in prevalence and outcome of many cardiac diseases [73]. However, much of our knowledge on cardiac disease is still based on studies done in males, and even today much experimental work in animal studies neglects females. The prevalence of dilated cardiomyopathy is higher in men, with both historic and current studies showing a ratio of 25–30% of women, compared with 70–75% men [3, 74–77]. Women are older at diagnosis by 1–3 years compared with men, and are more likely to present with heart failure and left bundle branch block [74–76]. However, outcomes are better for women, as both all-cause and cardiovascular mortality, as well as rates of heart transplants, malignant ventricular arrhythmias (MVAs), and sudden cardiac death are reduced in women compared to men [74–76]. Right ventricular function is also better in women compared to men [78]. However, as DCM is a heterogenous disease caused by a multitude of genetic defects and other causes, these results must not necessarily hold true for all sub-forms of DCM. In DCM caused by TTN truncating variants, men have a significantly worse prognosis [79]. In lamin A/C (*LMNA*) cardiomyopathy, one of the most arrhythmogenic forms of DCM and thereby comparable to RBM20 cardiomyopathy, end-stage heart failure, rates of MVAs, and mortality are increased in men [80]. RBM20 cardiomyopathy also presents in a more severe fashion in male patients: In a patient collective of 80 RBM20 mutation carriers, men were both younger at diagnosis and presented with lower ejection fraction (“age, 29 ± 11 versus 48 ± 12 years; *P* < 0.01; ejection fraction, 29 ± 13% versus 38 ± 9%; *P* < 0.01”) [17•]. One-third of male patients required a heart transplant, and at young ages (33 ± 16 years old), while none of the female patients required a heart transplant [17•]. A total event-free survival of male patients was also significantly reduced compared with female patients [17•]. Interestingly, male RBM20 cardiomyopathy patients also fared worse when compared to a cohort of patients with DCM of unknown genetic cause, while female RBM20 cardiomyopathy patients had better event-free survival when compared to patients with DCM of unknown cause, suggesting that these sex and gender differences are not shared by all forms of DCM, but rather (at least to some extent) specific to RBM20 cardiomyopathy [17•]. The cause of these sex and gender differences is so far unknown. Behavior-related risk factors differ in prevalence between the genders; for example, smoking is more common in men, while obesity has a higher prevalence in women [81, 82]. However, sex is likely also an independent risk factor. In fact, many cardiac genes show sexual dimorphism in their expression, which could change their interactions with RBM20’s splice targets and thereby affect heart function [83]. There are also large differences in gene expression between pre- and post-menopausal women, which might result in a loss of cardioprotective effects that could relate to the late onset of RBM20 cardiomyopathy in females [83]. Animal models of RBM20 cardiomyopathy may be useful to better understand these sex-specific genetic differences. One promising line of research is the altered calcium handling in RBM20 cardiomyopathy patients. The different prevalence of arrhythmias seems to be a major part of the differing outcomes, and other arrhythmogenic forms of DCM show similar differences [17•, 80]. Interestingly, CaMKIIδ might play a major part in this: As previously described, CaMKIIδ performs an isoform switch towards the CaMKIIδA and CaMKIIδ9 isoforms upon loss of RBM20 [11•]. Transgenic overexpression of CaMKIIδA alone is sufficient to induce a phenotype similar to RBM20 cardiomyopathy, and strikingly this largely affects male mice, leading to ~ 50% lethality at 8 weeks of age, while females are unaffected [16]. CaMKIIδ has already been shown to be sex-specifically regulated in cardiac disease: In ischemia-reperfusion experiments, female hearts showed higher generation of activated phospho-CaMKIIδB^(Thr287)^ and higher phosphorylation of its downstream target PLN, which was associated with arrhythmia suppression [84]. This might be due to differing expression of CaMK phosphatase (CaMKP), as CaMKP expression was increased in males and decreased in females after TAC surgery [85]. This cardioprotective effect of CaMKIIδB is unlikely to be the reason for gender differences in RBM20 cardiomyopathy, where CaMKIIδB is downregulated and the detrimental CaMKIIδA is much more common, but it shows that sex can affect the activity of specific CaMKIIδ isoforms [11•, 16]. It remains to be seen if CaMKIIδA and CaMKIIδ9 might also be differently activated. There is also evidence that calcium handling and excitation-contraction coupling in general are affected in a sex-dependent way. For example, female cardiomyocytes show lower calcium transients [86]. This may be caused by sex hormones, as testosterone can increase intracellular calcium and has been shown to be correlated with worse outcomes in other arrhythmogenic cardiac disease, while higher estrogen levels correlate with better outcomes [87, 88]. Likely, the observed gender differences are caused by a mixture of multiple genetic and non-genetic causes. Further research might not only advance treatment of RBM20 cardiomyopathy itself but also increase understanding of sex and gender differences in other forms of cardiac disease.

## RBM20 as a Therapeutic Target

Heart failure with preserved ejection fraction (HFpEF) is a highly relevant disease with a prevalence in patients aged 60 years or older of about 5% [89]. However, compared to heart failure with reduced ejection fraction (HFrEF), few effective therapeutic options exist [89, 90]. HFpEF is accompanied by changes in both titin and the extra-cellular matrix (ECM), which together contribute to an increase in myocardial stiffness [91]. Titin is differently phosphorylated at multiple sites: The S11878(S26) PKC/CaMKII site is hyperphosphorylated, while the S4185(S469) PKA/PKG site is hypophosphorylated, which together leads to a reduction of titin compliance, although the N2BA/N2B ratio does not change [91]. Additionally, the total amount of collagen in the heart is highly increased, which also increases myocardial stiffness [91]. The discovery of RBM20 as a highly effective modulator of titin-based stiffness opens up the possibility of using RBM20 as a treatment option both in HFpEF and other cardiac diseases that are be accompanied by a decrease in TTN compliance and an increase in myocardial stiffness [57–59]. Reducing Rbm20 activity has already shown positive effects, especially on diastolic function, in a mouse model with a deletion of Rbm20’s RRM [63, 92]. However, in otherwise healthy mice, the negative effects of reduced Rbm20 function are more pronounced; for example, the Frank-Starling mechanism is impaired and the mouse cannot fully compensate for cardiac stress [63]. More interesting and clinically relevant are studies on models for cardiac disease. In a model of HFpEF using transverse aortic constriction (TAC) surgery and deoxycorticosterone acetate (DOCA) pellet implantation, deletion of the RRM largely rescued the phenotype [64]. Functionally, this translated into an increased running distance after TAC [64]. Mechanistically, passive tension of cardiomyocytes (which is mainly determined by titin) decreased, while collagen-based stiffness was unaltered [64]. A similar rescue effect could also be observed in another HFpEF model with altered titin with increased passive tension [92]. Remarkably, in both cases, it was sufficient to suppress Rbm20 activity after the pathological state had already been established. However, as these models of HFpEF were mainly investigated in demembranated cardiomyocytes, they may not accurately represent the full phenotype of HFpEF [113]. In particular, they lack the contribution of cross-bridge mechanics, which have recently been shown to account for a significant amount of diastolic stiffness [115]. Therefore, further experiments on loss of RBM20’s effect on intact cardiomyocytes seem advisable, as the reduction of Ca^2+^ sensitivity caused by loss of RBM20 (in conjunction with adequate amounts of PKA activation) may counteract the increase in Ca^2+^ sensitivity which normally accompanies slowing of cross-bridge detachment [65, 113]. Currently, there exists one published drug discovery study on RBM20 splicing regulation, in which the group of cardenolides (including digoxin and digitoxin) is shown to specifically and potently be able to inhibit RBM20-dependent titin splicing [93]. Application of cardenolides led to a 10-fold reduction in RBM20 protein levels, and also changed the expression levels of several RBM20 interacting factors [93]. Cardenolides are already clinically used for cardiac disease; however, they have strict therapeutic levels, and potentially severe side effects. In other models, the loss of Rbm20 also reduced hypertrophy after volume overload and improved diastolic function and cardiac dimensions in a model with increased titin stiffness [56, 94]. Together, these findings suggest RBM20 manipulation as a valid therapeutic option, but reducing Rbm20 function also has negative effects. In general, heterozygous modifications of Rbm20 led to the best effects, while homozygous Rbm20 modifications came with an increase in negative side effects without corresponding benefits, like an increase in ECM stiffness and a worsened response to systolic stress [63]. The regulatory pathways affecting RBM20 are still poorly understood, and inhibiting RBM20 function might impact multiple pathways, not all of them in a beneficial way. For example, a 50% reduction in RBM20 already leads to potentially pro-arrhythmic calcium handling anomalies [11•]. Nevertheless, a greater understanding of how RBM20 functions and is regulated could open new avenues of treatment. For example, modulating RBM20’s PTMs, such as phosphorylation of the RSRSP stretch, could serve as a therapeutic target, especially in patients with mutations in the mutational hotspot. Another therapeutic approach would be the use of anti-sense oligonucleotides (AONs) that can bind and block RBM20 splicing sites, thereby reducing RBM20-dependent splicing [95]. AONs have already shown promising results in other diseases caused by altered splicing like Duchenne’s muscular dystrophy and spinal muscular atrophy [96, 97]. Interestingly, AONs can also be used to attract RNA-binding proteins or copy their function, which could be a therapeutic option for RBM20 cardiomyopathy patients [95, 98]. The use of small molecules to repress RBM20 splicing activity is also possible, although these may not only affect RBM20 [95, 99]. Different splice isoforms could also be directly targeted, with detrimental isoforms (such as CaMKIIδA) being knocked down using AONs, and protective isoforms being increased through the use of adeno-associated viruses [95]. In summary, RBM20 remains an intriguing target to therapeutically change titin (and thereby cardiac) compliance; however, potential side effects of RBM20 inhibition, including the potential induction of arrhythmias, cannot be neglected.

## Conclusion

Familial DCM is a heterogenous disease with different presentations depending on the genetic cause. However, treatment is currently not tailored to the specific genetic cause of DCM. RBM20 cardiomyopathy is an arrhythmogenic form of DCM, and early ICD implantation and anti-arrhythmic drug therapy may be fitting treatment options. A reduction of the cellular calcium overload, for example through the use of verapamil, may also lead to better outcomes for patients. In addition, male patients with RBM20 cardiomyopathy may need earlier and more intensive care, while female patients might be treated more conservatively. Understanding the cause of the gender-specific presentation of RBM20 cardiomyopathy will also allow better therapy for the heavily affected male patients. Eventually, therapies could focus either on a restoration of normal splicing through, for example, anti-sense oligonucleotides, or reintroduction of RBM20 itself through, for example, adeno-associated viruses. Much of the research on RBM20 so far has focused on its interactions with *TTN*, and while aberrant *TTN* splicing likely contributes to disease presentation, other avenues of research also should not be neglected.
